# Complications of percutaneous pedicle screw fixation in treating thoracolumbar and lumbar fracture

**DOI:** 10.1097/MD.0000000000011560

**Published:** 2018-07-20

**Authors:** Qinpeng Zhao, Haiping Zhang, Dingjun Hao, Hua Guo, Biao Wang, Baorong He

**Affiliations:** Department of Spine Surgery, Honghui Hospital, Xi’an Jiaotong University Health Science Center, Xi’an City, Shanxi Province, China.

**Keywords:** complications, minimally invasive, percutaneous pedicle screw, thoracolumbar and lumbar fracture

## Abstract

Percutaneous pedicle screw fixation (PPSF) has been a popular approach for treating thoracolumbar and lumbar fracture, and its relevant complications have been gradually recognized. This study aimed to summarize the complications of PPSF in treating thoracolumbar and lumbar fracture as well as the management and outcomes of the complications.

We retrospectively analyzed the patients with thoracolumbar and lumbar fracture who were admitted to our department from February 2011 to February 2015 and underwent posterior PPSF. Information on demographics, medical comorbidities, radiographs, and treatment was obtained from hospital medical records and follow-up records. Main outcome indexes included adverse clinical and radiological outcomes during and after surgery.

A total of 781 patients were included in this study. Forty-six patients (5.9%) presented with complications during or after surgery. The complications included intraoperative guide wire breakage, abdominal artery injury, spinal dura mater injury, postoperative pedicle screw misplacement, screw breakage, plug screw falling off, connecting rod loosening, poor reduction, and late infection. Among the 39 cases with postoperative complications, 14 underwent revision surgery, and the remaining patients underwent conservative treatment and presented good outcomes.

PPSF is associated with the following complications: guide wire rupture, blood vessel injury, cerebrospinal fluid leakage, screw misplacement, poor reduction, failed internal fixation, and infection. A thorough preoperative evaluation, accurate operation, and timely and correct management of complications are critical to achieving satisfactory surgical outcomes.

## 1 Introduction

Thoracolumbar and lumbar fracture is one of the most common types of spine injuries. Traditional treatment approaches often involve open internal fixation (OIF), which could result in great surgical trauma and blood loss and thus prolong the period of recovery.^[1–3]^ More recently, percutaneous pedicle screw fixation (PPSF) has been a popular approach for treating thoracolumbar and lumbar fracture, which could yield satisfactory outcomes.^[4,5]^ Compared with the OIF, PPSF does not require broad stripping and long-term traction of paraspinal muscles and has been associated with lower incidence of postoperative refractory backache and lumbar stiffness, less postoperative bleeding, and quicker recovery.^[6]^ Consequently, an increasing number of open surgeries are replaced by minimally invasive approaches. However, along with the broader acceptance of PPSF, relevant complications such as inadequate reduction and postoperative reduction loss have been gradually recognized.^[7–9]^ Currently, few studies have reported the incidence of the complications, relevant risk factors, and corresponding treatments. In this study, we retrospectively summarized the complications of PPSF, discussed the underlying cause of these complications, and the management and outcomes, which would be helpful for clinical decision-making.

## 2 Methods

### 2.1 Patient and information

This is a retrospective study of patients with thoracolumbar and lumbar fracture treated with PPSF at our spine surgery center from February 2011 to February 2015. This study was approved by the Institutional Review Broad, and all the patients provided signed informed consent. Inclusion criteria included the following: age from 17 to 60 years and patients who received PPSF for single level fracture involving T11 to L4. The exclusion criteria were as follows: surgical history of the level adjacent to the injured vertebra and patients with severe osteoporosis, spinal tumor, vertebral tuberculosis, or other suspected pathological fractures.

Information on demographics, medical comorbidities, radiographs, and treatment was obtained from hospital medical records and follow-up records. Postoperative x-ray and computed tomography (CT) examinations were performed to assess for vertebra reduction and screw position. Intraoperative, early and late postoperative complications were recorded.

### 2.2 Surgical procedure

Surgical procedures for the enrolled patients were performed by the same group of surgeons. After general anesthesia, the patient was placed in the prone position to maintain the thoracolumbar and lumbar level in a hyperextended position. C-arm x-ray machine was used to locate the incision site, and 4 2.0-cm longitudinal incisions were made. The puncture needle was placed at the inferior outer edge of the cortex projected by the vertebral pedicle, penetrated into the vertebral pedicle with manual rotation, and placed with its sagittal plane parallel to the adjacent endplates. Moreover, the needle was tilted inward (9°–15°) and was inserted into the vertebra via the pedicle. The inner core was pulled out and a guide wire was inserted. After confirming the proper location and depth of the guide wire, we inserted pedicle screws with a suitable size under the guidance of the guide wire and the placement was re-confirmed with the x-ray. The connecting rod was pre-bent into the physiological curvature after measuring its length and crossed each end of the screw successively with the assistance of a rod inserter to strut and reduce the injured vertebra. Normal saline was applied to clean the incision and thereafter the incision was sutured.

## 3 Results

A total of 781 patients were included in our study (465 men and 316 women), with the age ranging from 17 to 60 years old. Eighty-four cases of T11, 244 cases of T12, 238 cases of L1, 97 cases of L2, 72 cases of L3, and 46 cases of L4 fractures were involved. According to the AO classification, there were 272 cases of A1, 97 cases of A2, 249 cases of A3, 95 cases of A4, 49 cases of B1, and 19 cases of B2. The mean duration between injury and surgical intervention was 2.4 days (ranging from 1 to 5 days). None of the patients presented signs of spinal cord or nerve injury.

The mean operation duration was 63 minutes (ranging from 45 to 90 minutes), and the mean intraoperative bleeding volume was 80 mL (ranging from 50 to 110 mL). The mean follow-up duration was 22.9 months (ranging from 12 to 36 months). A total of 46 patients presented with complications (5.9%), including 7 cases during the operation, 21 cases early after the operation, and 18 cases late after the operation (Table 1).

**Table 1:**
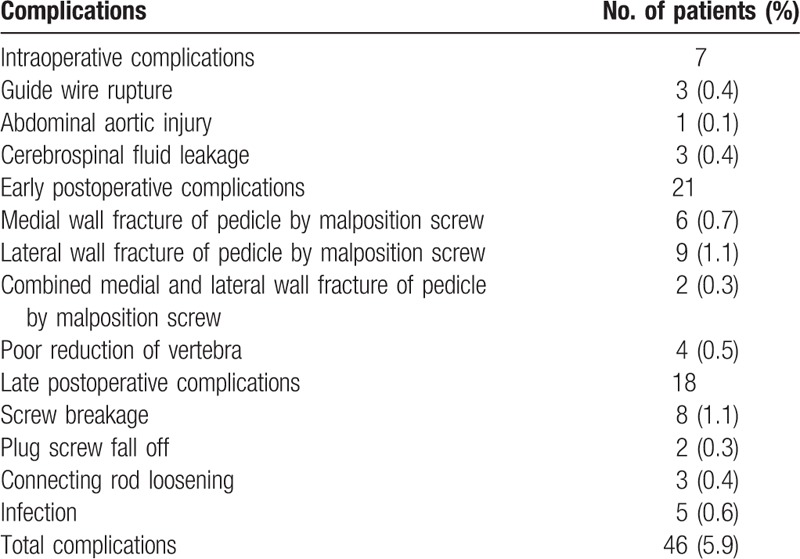
Complications associated with percutaneous pedicle screw fixation.

**Figure 1:**
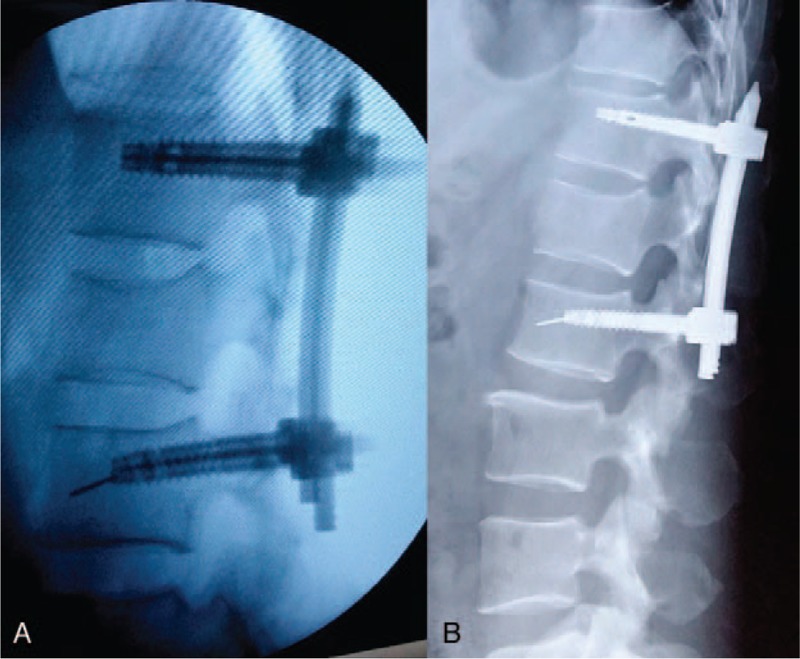
Male, 51 years old, L1 vertebral fracture (type A3): guide wire rupture during the operation (A); fracture healing achieved 1 year later, and guide wire was left in the vertebra (B).

**Figure 2:**
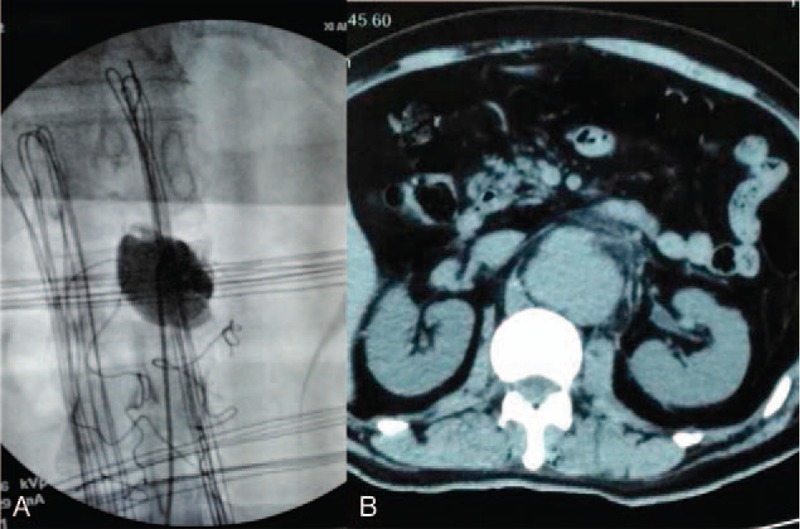
Male, 55 years old, L2 vertebral fracture (type A1): intraoperative angiography revealed abdominal aortic injury (A); postoperative CT showed pseudoaneurysm formation (B). CT = computed tomography.

Intraoperative complications included 3 cases of guide wire rupture: the guide wire was removed by pulling out the screw in 2 cases, and the guide wire could not be removed and was left in the vertebra permanently in 1 case (Fig. 1). One case of abdominal aortic injury was reported: the patient had intraoperative spray of blood and thus the surgery was terminated. Subsequently, intraoperative angiography was performed and revealed abdominal aortic injury and pseudoaneurysm formation. The patient was prescribed with follow-up (Fig. 2). Three cases of cerebrospinal fluid leakage due to screw canal enlargement with tapping were observed. After adjusting the screw canal and implanting the pedicle screw, the cerebrospinal fluid leakage could be stopped. The patients did not receive further treatment and presented good prognosis.

**Figure 3:**
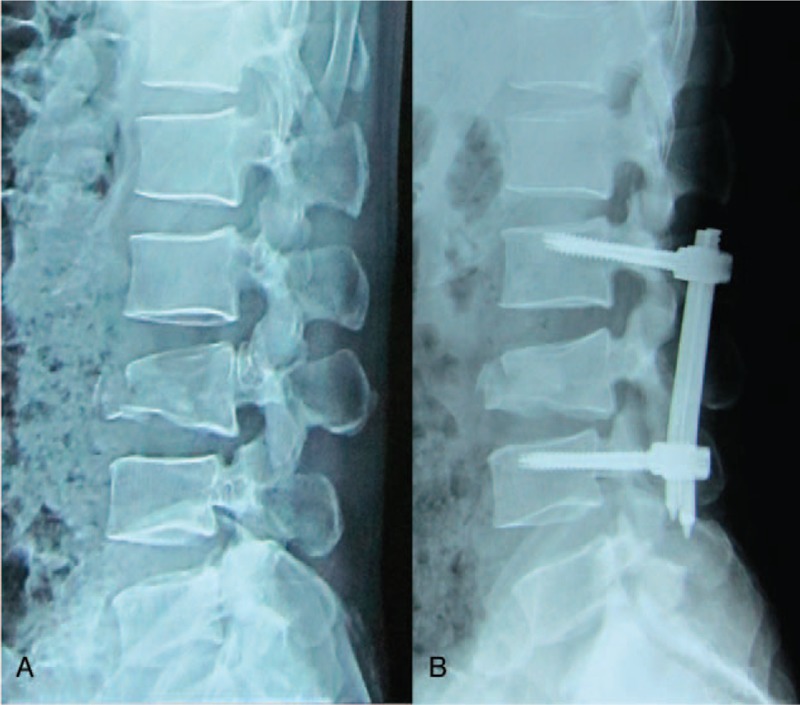
Male, 42 years old, L3 vertebral fracture (type B2): x-ray showed height loss and dislocation of L3 as well as increased spinous process distance (A); after percutaneous fixation, fracture reduction, and dislocation were not well corrected (B).

The early postoperative complications included 9 cases of single medial wall fracture of the pedicle, 6 cases of single lateral wall fracture of the pedicle, and 2 cases of combined medial and lateral wall fracture of the pedicle. Nine patients presented no signs of nerve injury and thus received no further treatment. Two patients presented neurological symptoms and underwent revision surgery to adjust the screw position. Patients with lateral wall fracture of the pedicle presented no signs of discomfort and were followed up, and they showed good outcomes. Postoperative x-ray films revealed 4 cases of poor reduction, which did not receive revision surgery (Fig. 3).

**Figure 4:**
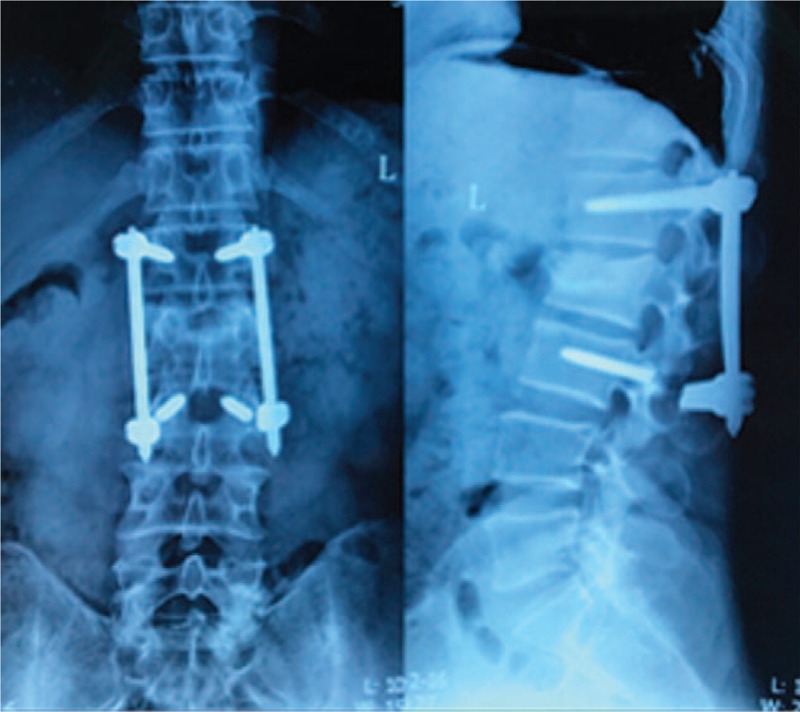
Male, 43 years old, L3 vertebral fracture (type A3): screw breakage at both sides of L3 8 months after surgery.

**Figure 5:**
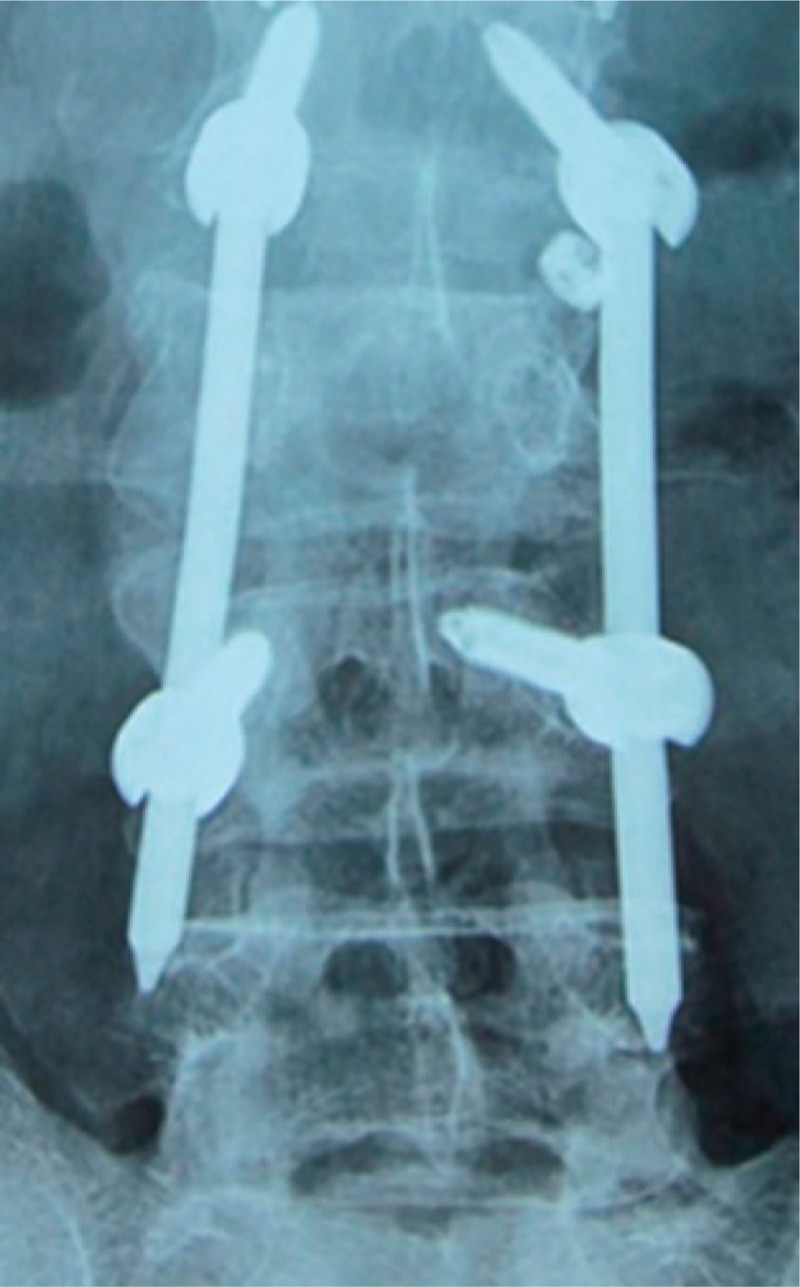
Female, 52 years old, L3 vertebral fracture (type A1): plug screw falling off at the left side of L2 3 months after surgery with connecting rod slipping down.

**Figure 6:**
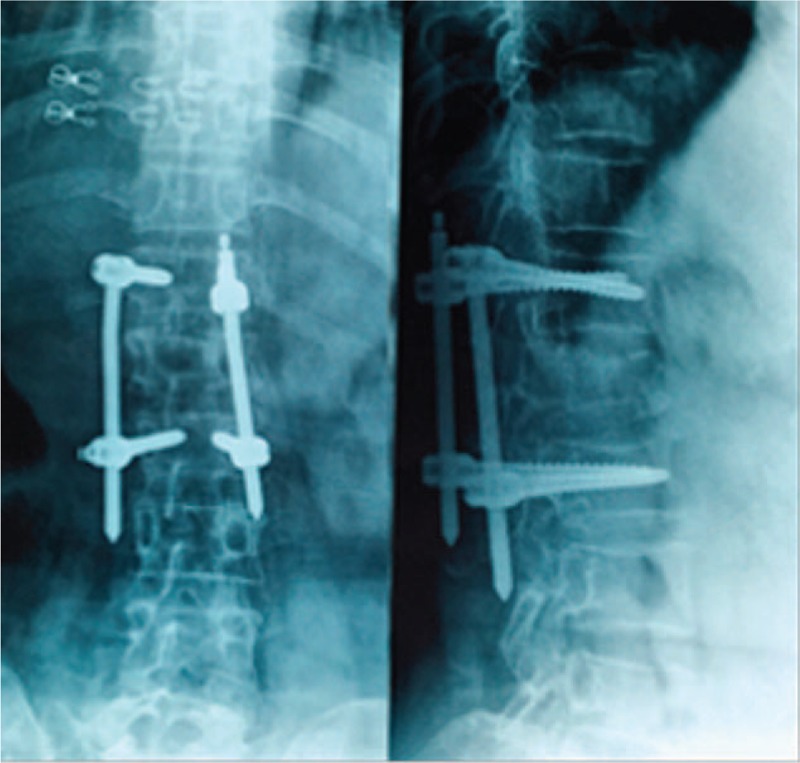
Female, 49 years old, L1 vertebral fracture (type A3): right connecting rod loosening and slipping 6 months after operation without plug off.

**Figure 7:**
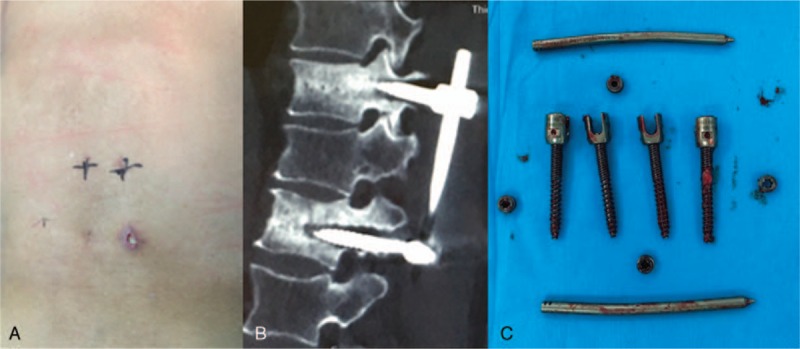
Female, 51 years old, T12 vertebral fracture (type A1): incision infection combined with sinus formation 10 months after operation (A); CT revealed fracture healing with screw loosening (B); removal of implants at revision surgery (C). CT = computed tomography.

The late postoperative complications included 8 cases of screw breakage (Fig. 4), and 7 of them occurred 4 to 6 months after operation. Among the 8 patients, 5 complained of discomfort and underwent revision surgery to reinforce the fixation screws, and after 1 year of follow-up, the patients presented good fracture healing; 2 patients showed no obvious discomfort, and the internal fixation was removed 12 months after operation; and 1 patient presented screw breakage 8 months after operation, and CT examination revealed fracture healing. Moreover, 2 cases of plug screw falling off were observed (Fig. 5), 1 of which occurred 3 months after operation and was treated with relocking of the screw under local anesthesia. The other case occurred 6 months after operation and received no special treatment (Fig. 6), and the implantation was taken out after fracture healing. In addition, there were 3 cases of connecting rod loosening, all of which occurred over 6 months after operation. Among them, 2 cases had internal fixation removal, and the other case that presented vertebral height loss received revision open surgery with posterolateral fusion. Five cases presented late deep infection 9 to 12 months after operation. They all presented fracture healing based on CT examination and underwent debridement and internal fixation removal under open surgery (Fig. 7). Pus was collected in all of the 5 patients for bacterial culture, and 1 case was positive for *Staphylococcus aureus*.

## 4 Discussion

Recently, PPSF technique has been developing rapidly. It could yield equal curative outcomes compared with OIF, but it is associated with minimal trauma, less bleeding, and quicker recovery.^[7]^ A prospective study with a 2-year follow-up that compared the outcomes of PPSF and OIF in treating thoracolumbar fractures showed that the radiographic and clinical outcomes were not significantly different between the 2 groups; however, the patients treated with PPSF showed a significantly shorter operative time, less blood loss, and less postoperative pain.^[10]^ Nonetheless, compared with OIF, PPSF also has drawbacks: due to the limited operation space, PPSF lacks anatomical markers, which could increase the risk of facet capsule injury. Complications of nerve injury and spinal dura mater rupture require revision open surgery. As PPSF requires special tools, surgical difficulty and the risk of complications like screw misplacement could be high, especially for surgeons without sufficient experience. High dose of x-ray and curve of education.

### 4.1 Complications and their causes

The guide wire breakage in our study could be due to the pedicle screw forming an angulation with the guide wire, which was truncated when driving the screws. Moreover, repeated use of the guide wire could also be one of the causes. Esses et al^[11]^ reported 1 case (0.16%) of blood vessel injury in 617 cases with OIF. In our study, 1 case of abdominal aortic injury (0.13%) was observed, which could be associated with misoperation during the screw position adjustment; the guide wire and screw were driven inward and injured the anterior wall of the vertebra, which in turn resulted in abdominal aortic injury. Moreover, although the C-arm machine and the surgical navigation system were used, it was still possible for the pedicle screws to penetrate the pedicle wall and enter the spinal canal, thus causing spinal dura mater injury and cerebrospinal fluid leakage.^[12]^ In our study, we noted 3 cases of cerebrospinal fluid leakage (0.40%) due to misoperation by the surgeons. Hence, a lack of anatomical markers during PPSF could be a major factor for the malposition of the screws.

A total of 3124 screws were implanted with the PPSF technique in our study. According to the Mobbs-Raley classification, which is used to assess the location of pedicle screws,^[11]^ a total of 17 cases had poor screw implantation (19 screws in total), which were associated with intraoperative misoperation and narrow vertebral pedicle (in 12 patients). Moreover, 4 cases with poor postoperative reduction were noted, 2 of which were misdiagnosed as having type C fracture with type B2 preoperatively. Magnetic resonance imaging examination revealed posterior ligament complex injury with dislocation and rotational injury. Once the connections between bone chips and ligament as well as disc annulus are lost, poor reduction could easily occur. In addition, with the wide application of polyaxial percutaneous pedicle screw, PPSF is associated with a high incidence of poor reduction.^[9,13,14]^

We also observed screw breakage, plug screw falling off, and connecting rod loosening in the postoperative follow-up. Short segment fixation without fixation of the injured vertebra could yield a great torque for the internal fixator and thus great stress, which could cause internal fixation loosening, breakage, and correction loss.^[15,16]^ Plug screw falling off could be mainly due to a tilted plug screw placement. Of the 5 cases of late infection in our study, 3 had diabetes, which is a risk factor of postoperative infection. According to a study by Jimenez-Mejias, 19.4% to 26% of postoperative spondylodiscitis is related to diabetes.^[17]^

### 4.2 Complication treatments and outcomes

For guide wire rupture, it is not necessary to take out the guide wire deliberately. Sticking to the standardized operation and applying guide wire made of memory alloys could greatly decrease the risk of guide wire breakage. For intraoperative blood vessel injury, the surgical procedure should be ceased immediately and intraoperative angiography must be performed to assess the aorta injury; moreover, intraoperative bleeding and vital signs need to be monitored closely during the operation, and multidisciplinary assistance to repair the vessels must be performed. For cerebrospinal fluid leakage, adjusting the position of pedicle screws after spinal dura mater injury could yield satisfactory outcomes. If cerebrospinal fluid leakage could not be alleviated, open revision surgery is recommended for spinal dura mater exploration and repair.

For patients with poor reduction, preoperative fracture type assessment must not be undervalued. Percutaneous short segment fixation should be avoided for patients with type C fracture. In addition, according to Kanna et al,^[18]^ from the point of injury due to vertebra fixation, the middle pedicle screws should be taken as the forward driving point to form a strong string force for reduction and correct kyphosis. Furthermore, single axis pedicle screws are superior to polyaxial pedicle screws in terms of fracture reduction.^[12]^

For failed internal fixation cases, patients with thoracolumbar discomfort that underwent open revision surgery with vertebra fixation in the early stage presented good outcomes. Patients without marked discomfort were prescribed with brace and regular re-examinations until good fracture healing and then the internal fixation was removed. In the late stage, the implants could be removed if fracture healing could be observed. Moreover, patients who presented with kyphosis after a failed internal fixation were treated with open surgical intervention with posterolateral fusion, which could well relieve pain and kyphosis. According to a study by Dobran et al,^[19]^ fixation via the injured vertebra could provide a satisfactory biomechanical correction and improve the rigidity of the pedicle screws and rods, thereby improving overall stress distribution, reducing local concentrated load, and avoiding screw breakage or loosening. For revision surgeries for screw breakage, rod breakage, or loosening, screw implantation at the injured vertebra would be an effective approach.

Five cases of late postoperative infection were found in our study, and all of the cases achieved fracture healing as confirmed with CT examinations. Revised open surgery was performed to remove the internal fixation followed by debridement, and no recurrent infection was observed. We believe that the key to controlling postoperative infection lies in early precaution and standardized operation. Treatment of concomitant internal diseases could also decrease the infection rate and improve the outcomes. The infection could be divided into early infection and late infection. For early deep spinal infection (occurring within 3 months of operation), debridement, and internal implant preservation are recommended.^[20]^ For late infection, internal implant preservation could yield high recurrence rate. Thus, we suggest removing the internal fixation at the first debridement.

This study has several limitations. This is a single-center retrospective study that excluded type C fracture; thus, the complication rate could be comparatively lower than that in open surgeries. In addition, our study lacks detailed case grouping and control study, and further prospective randomized case-control studies would be of great value to fully compare the minimally invasive approach with the open surgery approach.

In conclusion, PPSF in the treatment of thoracolumbar and lumbar fracture has merits of less surgical trauma and quicker recovery. However, it has risks of complications, such as guide wire rupture, blood vessel injury, cerebrospinal fluid leakage, screw misplacement, poor reduction of vertebra, failed internal fixation, and infection. Thorough preoperative evaluation, accurate operation, and timely and correct management of complications are crucial to achieving satisfactory surgical outcomes.

## Author contributions

**Conceptualization:** Qinpeng Zhao.

**Data curation:** Haiping Zhang, Hua Guo.

**Formal analysis:** Baorong He.

**Investigation:** Qinpeng Zhao, Haiping Zhang.

**Methodology:** Hua Guo, Biao Wang, Baorong He.

**Project administration:** Dingjun Hao.

**Resources:** Baorong He.

**Supervision:** Dingjun Hao.

**Validation:** Haiping Zhang, Hua Guo.

**Visualization:** Biao Wang.

**Writing – original draft:** Qinpeng Zhao, Haiping Zhang.

**Writing – review and editing:** Dingjun Hao.
